# Complete Genome Sequence of UV-Resistant *Campylobacter jejuni* RM3194, Including an 81.08-Kilobase Plasmid

**DOI:** 10.1128/genomeA.00305-16

**Published:** 2016-04-28

**Authors:** Nereus W. Gunther, Erin R. Reichenberger, James L. Bono

**Affiliations:** a U.S. Department of Agriculture, Agricultural Research Service, Wyndmoor, Pennsylvania, USA; b U.S. Department of Agriculture, Agricultural Research Service, Clay Center, Nebraska, USA

## Abstract

*Campylobacter jejuni* strain RM3194 was originally isolated from a human with enteritis and contains a novel 81,079-bp plasmid. RM3194 has exhibited superior survival compared to other *Campylobacter jejuni* strains when challenged with UV light. The chromosome of RM3194 was determined to be 1,651,183 bp, with a G+C content of 30.5%.

## GENOME ANNOUNCEMENT

Annually, *Campylobacter* spp. are responsible for the greatest number of foodborne gastrointestinal bacterial infections in the developed world (1, 2). However, *Campylobacter* spp. are nutritionally fastidious organisms requiring a microaerobic environment for survival (3). It remains uncertain by what mechanisms *Campylobacter* survives within multiple hostile environments in sufficient numbers to cause such significant amounts of human disease.

*Campylobacter jejuni* strain RM3194 was isolated from a clinical sample in 1994 from a human enteritis case at the Red Cross War Memorial Children’s Hospital in Cape Town, South Africa, and was supplied to our laboratory by Robert Mandrell (ARS, Albany, CA) (4, 5). In our research, RM3194 demonstrated an increased resistance to both UV (254 nm) and blue light (405 nm) (6). This resistance produced several-log-greater survival after challenge with UV light compared to the survival of other *C. jejuni* strains in our collection.

The genome of RM3194 and the large plasmid it contains was sequenced using a PacBio RSII system (Pacific Biosciences, Menlo Park, CA). Subread filtering was accomplished using the SMRT Analysis software suite, and assembly and error correction were performed using the Celera Assembler version 8.1 (7, 8). The resulting contigs were polished using the Quiver program, while the Geneious version 7.1.5 (Biomatters, Auckland, New Zealand) program was used to trim the overlapping ends of the contig and perform a reorientation. This was followed by a second application of Quiver to verify the previous trimming and reorienting steps (7). The assembly produced a coverage of 20× with a consensus accuracy of 99.9999%. The resulting closed RM3194 genome is composed of a single chromosome of 1,651,183 bp in size and a single large plasmid of 81,079 bp in size.

Given the observed resistance of RM3194 to UV light, it is interesting to note that the plasmid gene sequence with locus tag AXW77_08860 was very similar to a *C. jejuni* chromosomal gene that is believed to be part of a UV damage repair family of proteins (ImpB/MucB/SamB). It is therefore possible that this gene plays a role in the increased resistance to UV light demonstrated by RM3194. Additionally, the plasmid contains sequences that appear to be related to a complete bacterial type VI secretion system (AXW77_08625, AXW77_08630, AXW77_08635, AXW77_08640, AXW77_08645, AXW77_08650, AXW77_08990, AXW77_08995, AXW77_09000, AXW77_09005, AXW77_09010, AXW77_09015, and AXW77_09020), as well as sequences similar to other genes that permit conjugal-based plasmid transfer (AXW77_08765 and AXW77_08805) (9). This would suggest that this plasmid is capable of transferring between *Campylobacter* strains.

### Nucleotide sequence accession numbers.

The sequence of *C. jejuni* strain RM3194’s chromosome has been deposited in GenBank under accession no. CP014344 and the accompanying plasmid under accession no. CP014345.
